# The Influenza Virus Protein PB1-F2 Inhibits the Induction of Type I Interferon at the Level of the MAVS Adaptor Protein

**DOI:** 10.1371/journal.ppat.1002067

**Published:** 2011-06-09

**Authors:** Zsuzsanna T. Varga, Irene Ramos, Rong Hai, Mirco Schmolke, Adolfo García-Sastre, Ana Fernandez-Sesma, Peter Palese

**Affiliations:** 1 Department of Microbiology, Mount Sinai School of Medicine, New York City, New York, United States of America; 2 Institute of Global Health and Emerging Pathogens, Mount Sinai School of Medicine, New York City, New York, United States of America; 3 Department of Medicine, Mount Sinai School of Medicine, New York City, New York, United States of America; Johns Hopkins University - Bloomberg School of Public Health, United States of America

## Abstract

PB1-F2 is a 90 amino acid protein that is expressed from the +1 open reading frame in the PB1 gene of some influenza A viruses and has been shown to contribute to viral pathogenicity. Notably, a serine at position 66 (66S) in PB1-F2 is known to increase virulence compared to an isogenic virus with an asparagine (66N) at this position. Recently, we found that an influenza virus expressing PB1-F2 N66S suppresses interferon (IFN)-stimulated genes in mice. To characterize this phenomenon, we employed several *in vitro* assays. Overexpression of the A/Puerto Rico/8/1934 (PR8) PB1-F2 protein in 293T cells decreased RIG-I mediated activation of an IFN-β reporter and secretion of IFN as determined by bioassay. Of note, the PB1-F2 N66S protein showed enhanced IFN antagonism activity compared to PB1-F2 wildtype. Similar observations were found in the context of viral infection with a PR8 PB1-F2 N66S virus. To understand the relationship between NS1, a previously described influenza virus protein involved in suppression of IFN synthesis, and PB1-F2, we investigated the induction of IFN when NS1 and PB1-F2 were co-expressed in an *in vitro* transfection system. In this assay we found that PB1-F2 N66S further reduced IFN induction in the presence of NS1. By inducing the IFN-β reporter at different levels in the signaling cascade, we found that PB1-F2 inhibited IFN production at the level of the mitochondrial antiviral signaling protein (MAVS). Furthermore, immunofluorescence studies revealed that PB1-F2 co-localizes with MAVS. In summary, we have characterized the anti-interferon function of PB1-F2 and we suggest that this activity contributes to the enhanced pathogenicity seen with PB1-F2 N66S- expressing influenza viruses.

## Author Summary

Influenza viruses can cause global pandemics and are thus a major health concern. The novel H1N1 pandemic virus infected a large number of people, but resulted in relatively mild symptoms in the majority of cases. In contrast, the avian H5N1 viruses are associated with a high mortality rate, but are not transmitted from human to human. Understanding the viral and host factors that play a role in causing disease is crucial in developing effective vaccines and therapeutics. Furthermore, finding viral markers for high virulence may help predict the impact of newly emerging pandemic influenza viruses. We have previously established that a single amino acid substitution (N66S) in the viral PB1-F2 protein causes increased virulence in an H5N1 and the 1918 pandemic virus. Here we show that PB1-F2 N66S reduces the induction of interferon (IFN), a potent antiviral molecule secreted by cells in response to infection. Furthermore, we demonstrate that the inhibition of IFN by PB1-F2 N66S occurs at the level of the mitochondrial antiviral signaling protein (MAVS), a key player in the IFN production pathway. Our work here characterizes a new function for the PB1-F2 protein and how this function can lead to increased disease severity.

## Introduction

Influenza viruses cause annual epidemics and occasional pandemics which may result in up to 50 million excess deaths as seen during the outbreak in 1918 [1]. The virus strains causing the influenza pandemics that occurred in the years of 1918, 1957 and 1968, all have been found to express the virulence factor PB1-F2. The PB1-F2 protein is encoded by the +1 alternate open reading frame (ORF) in the PB1 gene of some influenza A viruses, giving rise to a 90 amino acid protein that was initially found to possess pro-apoptotic activity [2]. In animal studies, PB1-F2 has been shown to contribute to pathogenesis by delaying viral clearance, potentially by eliminating immune cells via apoptosis [3]. A recent study has identified a single point mutation at amino acid position 66 in the PB1-F2 protein of the 1918 pandemic and of an H5N1 influenza virus strain, which is associated with increased virulence [4]. This mutation consists of a change of the amino acid asparagine (66N) to serine (66S) and causes increased weight loss and viral loads in infected mice. In search of the molecular mechanism for these findings, microarray analyses on whole lung homogenates of mice infected with isogenic viruses expressing either PB1-F2 66N or 66S were performed. Interestingly, infection with a virus expressing PB1-F2 N66S lead to a suppression of interferon-stimulated genes (ISGs) at an early stage of infection [5]. In the present study, we aim to verify these findings *in vitro* and elucidate the molecular mechanism for the IFN antagonism function of PB1-F2 N66S.

The innate immune system is the first line of defense in response to viral infection. Besides Toll-like receptors (TLR) and Nod-like receptors (NLRs) in the endosome and cytoplasm, respectively, RNA helicases such as the retinoic acid inducible gene-I (RIG-I) and the melanoma differentiation-associated gene-5 (MDA-5) are able to recognize characteristic patterns of invading pathogens and induce the production of type I interferons (IFN), potent antiviral molecules [6], [7]. In influenza virus infected cells, RIG-I has been shown to be the major sensor of viral RNA leading to type I IFN production, as knock down of RIG-I expression has been demonstrated to abolish type I IFN secretion in response to virus infection [8]. Expression of type I IFN genes has been found to be regulated by the so-called enhanceosome, constituted by the transcription factors IRF3/7, NF-κB and ATF/c-Jun [9]. Upon recognition of viral RNA species, RIG-I interacts with the mitochondrial antiviral signaling protein (MAVS, also known as IPS-1, VISA, CARDIF) in the mitochondrial membrane. This leads to the phosphorylation and activation of both IRF3 and IRF7 by IKKε and TBK1 [10]. Upon secretion, IFN binds to specific IFN receptors in an autocrine or paracrine manner and activates the JAK/STAT pathway. This leads to the formation of the ISG factor 3 (ISGF3) transcription complex which drives the expression of antiviral genes such as protein kinase R (PKR), Mx GTPases and others.

Viruses have evolved mechanisms to counteract the antiviral IFN production and/or signaling pathways. Nipah virus V protein has been shown to bind STAT1 and thus inhibit nuclear translocation of ISGF3 [11], [12]. The VP35 protein of Ebola virus can block IFN induction by interacting with both TBK1 and IKKε and interfere with the phosphorylation of IRF3 [13]. Influenza virus expresses the non-structural protein 1 (NS1) which can inhibit IFN induction using multiple strategies [14]. NS1 has been shown to bind dsRNA and thus mask viral RNA species from recognition [15], [16]. Furthermore, NS1 interacts with RIG-I and its co-activator TRIM25 leading to impaired activation of the IRF3, ATF/c-Jun and NF-κB transcription factors that drive IFN-β expression [17], [18], [19], [20]. In addition, NS1 can interact with PKR and inhibit its activation [21], [22]. Recent reports describe an IFN antagonism function also for PB2 and other polymerase proteins [23], [24]. In the study by Graef *et al.* [23], PB2 was found to interact with MAVS at the mitochondria and thus impair IFN-β production without apparently affecting viral replication *in vitro*.

Besides NS1, PB1-F2 is another non-structural protein of influenza viruses [25]. In contrast to NS1, the molecular mechanism for the contribution of PB1-F2 to the pathogenicity of influenza virus has not been thoroughly studied. PB1-F2 triggers the intrinsic apoptosis pathway by interacting with the mitochondrial adenine nucleotide translocator 3 (ANT3) and voltage-dependent anion channel 1 (VDAC1) proteins *in vitro* [26] and it induces apoptosis in a strain-specific way [27]. Several studies demonstrated that PB1-F2 has pro-inflammatory activity and exerts its function as a virulence factor by causing increased immune cell infiltration, elevated cytokine levels and tissue damage [4], [27], [28]. A study by Mazur *et al.* describes an interaction of PB1-F2 with the PB1 subunit of the viral polymerase complex in virus infected cells, but this interaction does not seem to contribute to pathogenicity in a mouse model [29], [30].

In search for residues in the PB1-F2 protein that confer increased virulence, we have previously identified a serine at position 66 that is associated with increased pathogenicity by suppressing the early IFN response in a mouse infection model [4], [5]. Herein, we confirm and characterize the IFN antagonism function of PB1-F2 *in vitro*, finding that a serine at position 66 enhances the anti-IFN activity in an overexpression system, when expressed from a Newcastle disease virus (NDV) vector as well as in the context of influenza virus infection. Furthermore, we show that PB1-F2-mediated IFN suppression is exerted via its C-terminal domain at the level of the MAVS adaptor protein. We also investigated the relationship between PB1-F2 and the well characterized IFN antagonist NS1 and observed that PB1-F2 N66S in combination with NS1 lead to lower IFN induction compared to NS1 alone.

Based on our results we propose that PB1-F2 proteins of highly pathogenic influenza virus strains contribute to pathogenesis by suppressing the host innate response at the level of the MAVS adaptor protein. This is the first report that links a molecular mechanism to the observed pathogenic phenotype caused by PB1-F2 *in vivo*.

## Materials and Methods

### Ethics statement

This study was carried out in strict accordance with the recommendations in the Guide for the Care and Use of Laboratory Animals of the National Institutes of Health. The protocol was approved by the Institutional Animal Care and Use Committee at Mount Sinai School of Medicine (Permit Number: 03-0058). Mice were sacrificed for the isolation of bone marrow according to these guidelines and all efforts were made to minimize suffering.

### Cell lines and antibodies

Madin Darby Canine Kidney (MDCK), 293T, Vero and A549 cells were obtained from ATCC (Manassas, VA, USA) and were maintained in Minimal Essential Medium (MEM) or Dulbecco's Modified Eagle Medium (DMEM) (Gibco, Invitrogen, San Diego, CA, USA) supplemented with 10% fetal bovine serum (FBS, Hyclone, South Logan, UT, USA) and penicillin/streptomycin (Gibco). LA-4 cells were obtained from ATCC and maintained in F-12K medium (Gibco) supplemented with 15% FBS (Hyclone) and penicillin/streptomycin (Gibco). The generation of the MDCK cell line constitutively expressing the IFN-β reporter (MDCK-IFN-beta luc) has been described before [31] and this cell line was maintained in DMEM with 10% FBS (Hyclone) and penicillin as well as hygromycin and geneticin (Gibco). Monoclonal antibodies to actin, FLAG and HA were obtained from Sigma-Aldrich (St.Louis, MO, USA). NP protein levels were detected using a monoclonal NP antibody generated by our laboratory (clone 28D8). The polyclonal rabbit NS1 antibody was raised against amino acids 1–73 of the Tx/98 swine virus NS1 protein. The polyclonal rabbit sera against PB1-F2 has been described before [26]. The polyclonal rabbit serum against NDV was prepared by Dr. Qinshan Gao.

### Isolation and culture of primary human and murine dendritic cells (DCs)

Peripheral blood mononuclear cells were isolated by Ficoll density gradient centrifugation (Histopaque, Sigma- Aldrich) from buffy coats of healthy human donors (New York Blood Center) as previously described [32]. Briefly, CD14-positive cells were immunomagnetically purified using anti-human CD14 antibody-labeled magnetic beads and iron-based MiniMACS LS columns (Miltenyi Biotec, Auburn, CA, USA). After elution from the columns, cells were plated (10^6^ cells/mL) in DC medium (RPMI medium [Invitrogen], 2 mM L-glutamine [Invitrogen], 1 mM Sodium Pyruvate [Invitrogen] and penicillin-streptomycin [Invitrogen] and 10% FBS [Hyclone]) supplemented with 500 U/mL human granulocyte macrophage colony-stimulating factor (GM-CSF, Peprotech, Rocky Hill, NJ, USA), and 1,000 U/mL human interleukin-4 (IL-4, Peprotech) and incubated for 5 days at 37°C.

Murine dendritic cells were obtained from bone marrow as described previously [33]. Bone marrow was prepared from the leg bones of 7–9 week old BL/6 mice (Jackson Laboratories, Bar Harbor, ME, USA). Tibia and femur were aseptically dissected and the bone marrow flushed out. Bone marrow cells were cultured with Iscove's modified Dulbecco's medium (Gibco) supplemented with GM-CSF (20 ng/mL; Peptrotech), non-essential amino acids (Gibco), 50 mM β-mercaptoethanol (Gibco), 10% FBS (Hyclone) and penicillin-streptomycin (Gibco) at 37°C in 5% CO_2_ for 7 days. Fresh media was added every second day. Floating dendritic cells were recovered and seeded for subsequent viral infection the following day.

### Rescue of recombinant influenza and Newcastle disease viruses

The influenza A/Puerto Rico/8/1934 (PR8; H1N1) viruses were rescued as described previously [34]. Briefly, 293T cells were transfected with seven bidirectional pDZ constructs for PB2, PA, NP, HA, NA, M and NS (giving rise to viral genomic and messenger RNAs) as well as a pPolI construct expressing the wildtype (WT) or the PB1-F2 N66S PB1. In addition, four pCAGGS protein expression vectors encoding the subunits of the WSN viral polymerase and the nucleocapsid protein were added. The transfected 293T cells were injected into 10-day old embryonated chicken eggs (Charles Rivers Laboratories, Wilmington, MA, USA) and propagated for 48 h. Rescued viruses were plaque purified on MDCK cells, propagated in 10-day old embryonated chicken eggs and sequenced to confirm the presence of the introduced mutations. The N66S mutation in the PB1-F2 open reading frame was introduced by a single point mutation at position 315 in the PB1 gene which changed the nucleotide from an A to a G using the Stratagene Quick-Change mutagenesis kit (Stratagene, La Jolla, CA, USA). This mutation does not affect the PB1 amino acid sequence. For rescue of the recombinant PR8 virus expressing a dsRNA/TRIM25 binding mutant form of NS1 (R38A/K41A), a pCAGGS-NS1 expression plasmid has been added to the 293T transfection mixture and 7-day old embryonated chicken eggs (Charles Rivers Laboratories) were used as described before [20].

The PB1-F2 (WT and N66S, PR8) expressing Newcastle disease viruses (NDV) were generated from the LaSota strain cDNA as described before [35], [36]. The PB1-F2 and GFP genes were inserted into the NDV genome between the P and M segments via Sac II restriction sites. The NDV-GFP virus was prepared by Dr. Qinshan Gao [37] . The NDV-NS1 (B1 strain) virus has been described previously [32].

### Infection of cells with NDV or influenza viruses

Primary human DCs were infected with the NDV recombinant viruses at a multiplicity of infection (MOI) of 2 or the NS1 dsRNA/TRIM25 binding mutant PR8 influenza viruses at an MOI of 0.5 in serum-free DC media for 45 min at 37°C. After the adsorption time, DCs were plated in complete DC medium and incubated for indicated timepoints at 37°C. Then, cells were recovered by centrifugation at 400× g for 10 min for subsequent RNA isolation. In addition, supernatants were collected for cytokine production evaluation by Multiplex ELISA (Millipore, Billerica, MA, USA). Adherent cells were washed with PBS and virus diluted in PBS supplemented with 0.3% bovine albumin (BA, Gibco) and penicillin streptomycin (Gibco) was added for 1 h at 37°C. Cells were subsequently washed and growth media was added in case of single cycle analyses. For multicycle analyses, DMEM (Gibco) supplemented with 0.1% FBS (Hyclone) and 0.3% BA (Gibco) including 1 µg/mL trypsin was added.

### RNA isolation

RNA from human DCs (5×10^5^) was extracted using the Absolutely RNA Microprep Kit (Stratagene). RNA yields were evaluated in a Nanodrop spectrophotometer (Nanodrop technologies, Wilmington, DE, USA) at 260 nm, and 500 ng of RNA was reverse transcribed using the iScript cDNA Synthesis Kit (Bio-Rad, Hercules, CA, USA) according to the manufacturer's instructions. RNA from all other cells was harvested using β-mercaptoethanol-containing RLT buffer from the RNeasy Qiagen kit and RNA was extracted following the manufacturer's instructions; cDNA was generated using the Superscript First-Strand RT-PCR kit (Invitrogen) according to the manufacturer's instructions.

### Quantitative real- time PCR (qRT-PCR)

Evaluation of mRNA levels was carried out using iQ SYBR Green SuPermix (Bio-Rad) according to the manufacturer's instructions. The PCR temperature profile was 95°C for 10 min, followed by 40 cycles of 95°C for 10 s, 60°C for 60 s. The mRNA levels of target genes were normalized to α-tubulin and rps11 expression for human DCs and 18S for all other cell types, respectively. The sequences of the primers used for qRT-PCR analyses on DC samples have been described elsewhere [38]. The primers for viral M and PB1 RNA levels detect cRNA, vRNA and mRNA and the sequences are as follows: 5′-TCAGGCCCCCTCAAAGCCGA-3′ (forward) and 5′-GGGCACGGTGAGCGTGAACA-3′ (reverse) for M, 5′-AATTCTTCCCCAGCAGTTCA-3′ (forward) and 5′-TTTTTGCCGTCTGAGCTCTT-3′ (reverse) for PB1. The primers used to quantify murine IFN-β are 5′-CAGCTCCAAGAAAGGACGAAC-3′ (forward) and 5′-GGCAGTGTAACTCTTCTGCAT-3′ (reverse). The primers for murine IP-10 are: 5′-TTCACCATGTGCCATGCC-3′ (forward) and 5′-GAACTGACGAGCCTGAGCTAGG-3′ (reverse). The primers used for A549 cells are: 5′-TCTGGCACAACAGGTAGTAGGC-3′ (forward) and 5′-GAGAAGCACAACAGGAGAGCAA-3′ (reverse) for IFN-β, 5′-GTAACCCGTTGAACCCCATT-3′ (forward) and 5′-CCATCCAATCGGTAGTAGCG-3′ (reverse) for 18S and 5′-GGAACCTCCAGTCTCAGCACCA-3′ (forward) and 5′-AGACATCTCTTCTCACCCTTC-3′ (reverse) for IP-10. All experiments included biological triplicates and technical duplicates. CXF Manager software (Bio-Rad) was used to analyze the normalized relative mRNA levels in the samples.

### Cytokine quantification in human DC supernatants

The levels of IFN-α and IP-10 proteins in human DC supernatants after infection was quantified using the MILLIPLEX Multi-Analyte Profiling Human Cytokine/Chemokine Kit (Millipore) according to the manufacturer's instructions. Data were analyzed using the Milliplex Analyst Software (Millipore).

### Expression plasmids and cloning

The pCAGGS vector possessing a chicken β-actin promoter has been described previously [39]. An N-terminal FLAG tag was added to PB1-F2 WT and N66S (PR8) as well as NS1 genes (PR8) by PCR with 5′ gene-specific primers containing the tag sequence and cloned into pCAGGS using EcoRI and XhoI restriction sites. The HA-tagged Nipah virus V protein, the FLAG-tagged Ebola virus VP35 protein, FLAG-tagged RIG-I N, HA-tagged MAVS, FLAG-tagged TRIF, FLAG-tagged TBK1 and IKKε, IRF3-5D expression plasmids have been described elsewhere [13], [40]. The generation and subcellular localization of the PB1-F2 truncation constructs have been described previously [26].

### Growth curves of recombinant viruses

Ten-day old embryonated chicken eggs (Charles Rivers Laboratories) were inoculated with 100 plaque forming units (PFU) of virus and incubated at 37°C for the indicated amount of time and subsequently placed at 4°C. Allantoic fluids were harvested and centrifuged at 3,000 rpm for 30 min at 4°C. A549 cells were infected at a multiplicity of infection (MOI) of 0.01 and supernatants were harvested at the indicated time points and centrifuged at 1,200 rpm for 5 min to remove cell debris. The viral titers were determined via plaque assays on MDCK cells.

### NDV-GFP bioassay

The NDV-GFP bioassay to quantify IFN levels has been described before [12]. Briefly, 293T cell supernatants were harvested and spun down at 1,200 rpm for 5 min to remove cell debris. Vero cells grown in a 96-well format were overlaid with serial 2-fold dilutions of the supernatants for 24 h and subsequently infected with NDV-GFP at an MOI of 5. The fluorescence intensities were measured with a plate reader (Beckman Coulter DTX 880 instrument) at 18–24 hours post infection (hpi) (excitation wavelength: 485 nm, emission wavelength: 535 nm). Images were taken using a fluorescence microscope (Olympus IX70).

### Reporter assays

For IFN-β reporter assays, 293T cells were transfected with lipofectamine 2000 (Invitrogen) at a ratio of 1∶1 with plasmid DNA and lysed 24 h post transfection. For the ISRE reporter assay, transfected 293T cells were stimulated with 1000 U/mL of universal type I interferon (PBL interferon source, Piscataway, NJ, USA) and lysed 24 h later. For luciferase assays, cells were lysed using the provided lysis buffer by the dual-luciferase assay kit according to the manufacturer's instructions (Promega, Madison, WI, USA). The fold-induction was calculated as the ratio of stimulated versus unstimulated samples. Expression plasmids for RIG-I N, MAVS, TBK1, IKKε, IRF3-5D and TRIF were transfected as stimuli for the IFN-β reporter assays.

### Western blot analysis

Cells were lysed in urea buffer (6 M urea, 2 M β-mercaptoethanol, 4% sodium dodecyl sulfate [SDS]) and sonicated three times at output level 3.0 for 5 s. Samples were run on 4–20% precast gradient gels (Bio-Rad) and transferred onto polyvinylidene fluoride (PVDF) membranes (GE Healthcare, Buckinghamshire, UK). Blotting against HA or FLAG-tagged proteins as well as PB1-F2, actin and viral proteins was achieved by using antibody dilutions of 1∶1000 in 5% non-fat dry milk-containing PBS-Tween-20 0.05%. Anti-NP, NS1 and NDV antibodies were used at a dilution of 1∶2000 and 1∶5000, respectively. Horseradish peroxidase-conjugated secondary antibodies (GE Healthcare) were used at a dilution of 1∶5000.

### Confocal microscopy studies

Hela cells were transfected as described above for 293T cells to overexpress HA-tagged MAVS, FLAG-tagged PB1-F2 or empty vector and allowed to adhere to round glass slides in a 24-well plate. Twenty-four h post transfection, cells were fixed with 4% para-formaldehyde and stained with anti-FLAG and anti-HA antibodies as well as DAPI. Secondary antibodies conjugated to Alexa 488 and Alexa 555 (Invitrogen) were used to visualize the proteins. Images were taken on an LSM 510 Meta confocal microscope (Carl Zeiss MicroImaging GmbH, Jena, Germany) at a magnification of 100×. Confocal laser scanning microscopy was performed at the MSSM-Microscopy Shared Resource Facility, supported with funding from NIH-NCI shared resources grant (5R24 CA095823-04), NSF Major Research Instrumentation grant (DBI-9724504) and NIH shared instrumentation grant (1 S10 RR0 9145-01).

## Results

### PB1-F2 overexpression impairs RIG-I N induced type I IFN production

**Figure 1:**
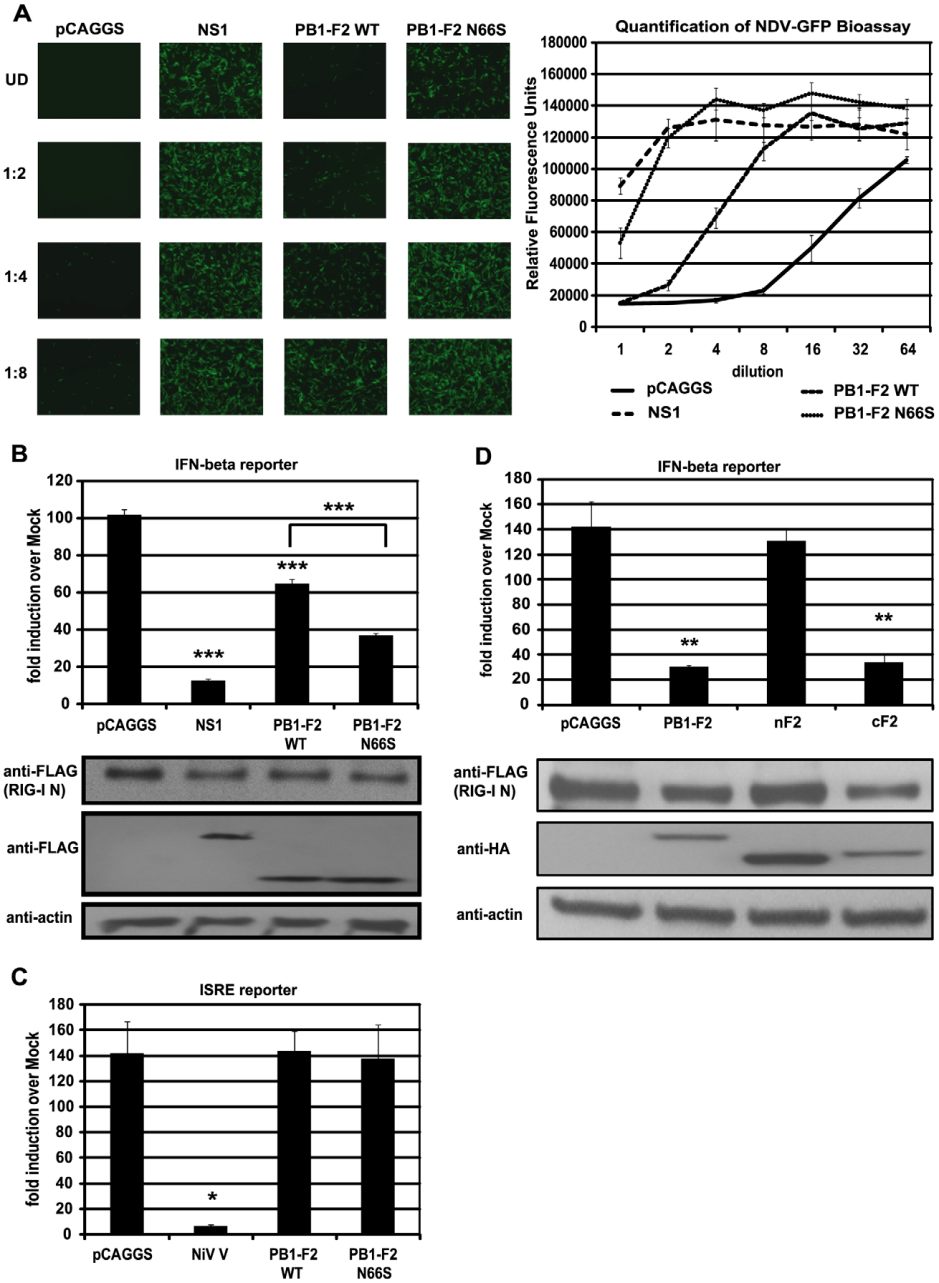
PB1-F2 inhibits RIG-I N induced IFN via its C-terminal domain. (A) IFN secretion was determined via NDV-GFP bioassay. 293T cells were transfected with empty vector (pCAGGS), NS1 or PB1-F2 WT or N66S expressing vectors as well as a RIG-I N expression plasmid. Twenty-four hours post transfection, supernatants were harvested and serial two-fold dilutions of the supernatants were used to overlay Vero cells. Twenty-four hours later, Vero cells were infected with NDV-GFP and images were taken 18 hpi. To quantify the GFP signals, the plate was analyzed by a plate reader with an excitation wavelength of 485 nm and an emission wavelength of 535 nm. (B) 293T cells were transfected as described in (A) with the addition of IFN-β and renilla reporter constructs. Twenty-hour hours post transfection, cells were harvested and luciferase and renilla signals were determined by luminometry. In addition, cells were harvested for Western blot analyses. (C) 293T cells were transfected as described in (A) with the addition of ISRE and renilla reporter constructs. Twenty-hour hours post transfection, cells were treated with 1,000 U/mL of universal type I IFN. Twenty-four hours after IFN treatment, cells were harvested for luminometry and Western blot analyses. (D) 293T cells were transfected and analyzed as described in (B). Besides full-length PB1-F2, following truncation constructs were tested for IFN antagonism activity: the N-terminal domain consisting of amino acids 1–37 (nF2) and the C-terminal domain spanning amino acids 38–87 (cF2). All constructs carried an HA tag and were fused to GFP. All data represent means ± standard deviations of one representative experiment (n = 3). Statistical significance was determined using Student's *t* test. *, p<0.05; **, p<0.01; ***, p<0.001. All data shown are representatives of at least three independent experiments.

In our previous study we have shown that an influenza virus expressing PB1-F2 66S of an H5N1 virus suppresses interferon-stimulated genes (ISGs) at an early stage of infection *in vivo* compared to an isogenic virus expressing PB1-F2 66N [5]. To confirm and characterize the interferon (IFN) antagonism function of PB1-F2, we employed several *in vitro* assays. We first examined whether PB1-F2 could block IFN secretion induced by constitutively active RIG-I, since RIG-I is the most upstream molecule involved in triggering the antiviral IFN response against influenza virus. We transfected 293T cells, which are highly transfectable, with a plasmid expressing constitutively active RIG-I (RIG-I N) as well as an empty vector (pCAGGS), NS1 (PR8) or PB1-F2 (PR8) WT or N66S expressing constructs and measured IFN secretion by these cells in an NDV-GFP bioassay. Vero cells were overlaid with 293T cells supernatants and infected with NDV-GFP virus 24 hours later. The replication efficiency of the NDV-GFP virus is a measure for the amount of IFN in the supernatants where a high GFP signal indicates IFN suppression activity and low GFP expression is a read-out for high IFN concentrations. Expression of an empty vector did not block RIG-I N induced IFN secretion, whereas the well described IFN antagonist NS1 enabled NDV-GFP to replicate efficiently (Figure 1A). Interestingly, overexpression of PB1-F2 also blocked IFN production and a serine at position 66 (N66S) showed an increased IFN antagonism activity in the NDV-GFP bioassay (Figure 1A). We did not observe any cytopathic effects by expressing PB1-F2 proteins without any additional stimuli which is in accordance with previous findings [26].

Of note, the NDV-GFP bioassay measures not only the amount of secreted IFN, but also the activity of other antiviral molecules. Hence, to examine whether PB1-F2 interferes with the activation of the IFN-β promoter, we performed an IFN-β reporter assay, using a reporter construct that carries the IFN-β promoter driving the expression of a firefly luciferase gene. This reporter was transfected into 293T cells along with a constitutively expressed renilla luciferase reporter control to monitor cytotoxicity and transfection efficiency. As shown in Figure 1B, overexpression of RIG-I N strongly induced the reporter (approximately 100-fold) which was not affected by the empty vector control (pCAGGS), but was efficiently blocked by NS1. In accordance with the bioassay data, PB1-F2 decreased activation of the IFN-β reporter and PB1-F2 N66S was approximately two-fold more efficient in inhibiting the reporter compared to PB1-F2 wildtype (WT) at equal protein amounts (Figure 1B). Compared to NS1, PB1-F2 WT inhibited IFN induction about four times less efficiently, whereas PB1-F2 N66S was only two-fold less efficient than NS1 in these assays (Figure 1A and 1B). We also tested the influenza virus nucleoprotein (NP) and observed no inhibition of the RIG-I N induced IFN-β reporter (data not shown). To examine whether PB1-F2 proteins from other strains besides PR8 could inhibit IFN induction, we tested both PB1-F2 66N and 66S proteins from the A/Brevig Mission/1/1918 (H1N1) pandemic and A/Viet Nam/1203/2004 (H5N1) strains and found similar results (data not shown). Activation of RIG-I leads to the secretion of type I IFN which binds to IFN receptors to activate the JAK/STAT pathway resulting in the establishment of an antiviral state in a paracrine or autocrine manner. To examine whether PB1-F2 is also able to interfere with IFN signaling, we employed an interferon-stimulated response element (ISRE) reporter assay that contains an ISG54 promoter fused to a firefly luciferase gene. 293T cells were transfected with ISRE and renilla reporters as well as an empty vector, PB1-F2 plasmids or a vector expressing Nipah virus V protein as a positive control [11]. Twenty-four hours post transfection, 293T cells were treated with universal type I IFN and analyzed for reporter activity 24 hours later. Nipah virus V protein strongly repressed activation of the ISRE reporter, whereas empty and PB1-F2 expressing vectors did not affect the ISRE reporter (Figure 1C). We have also not observed an effect of PB1-F2 on the ISRE reporter in unstimulated cells (data not shown).

### The C-terminal domain of PB1-F2 mediates its IFN antagonism activity

It has been demonstrated that a C-terminal portion of PB1-F2 interacts with both ANT3 and VDAC1 to induce apoptosis, while an N-terminal fragment of PB1-F2 is not able to trigger cell death [26]. In view of this finding, we tested whether the IFN antagonism function of PB1-F2 was confined to the N- or C-terminal domain. For this purpose, we used an N-terminal fragment of PB1-F2 which contains amino acids 1–38 and a C-terminal portion that contains amino acids 39–87 of the PR8 PB1-F2 protein. The design of the PB1-F2 truncations is based on previous structural data by Bruns *et al.* [41]. Of note, the C-terminal domain includes the mitochondrial localization sequence (MLS). These PB1-F2 fragments were fused to GFP for protein stability and also contain an N-terminal HA tag as described previously [26]. As shown in Figure 1D, the C-terminal fragment and the full-length PB1-F2 protein which is also fused to GFP and contains an HA tag, inhibited the IFN-β reporter to similar levels. Conversely, the N-terminal portion was unable to block the reporter even though it was expressed at higher levels than the other constructs. It is possible that localization of these peptides to the mitochondria is necessary for the IFN antagonism function, so we also tested an N-terminal PB1-F2 fragment that contains an MLS derived from the human cytochrome C oxidase as described by Zamarin *et al.* [26]. Even though this peptide localizes to the mitochondria as efficiently as the full-length PB1-F2 construct [26], it was still unable to suppress IFN induction in this assay (data not shown). Collectively, these results suggest that the C-terminus, which contains the characteristic amphihelical structure, mediates the IFN antagonism function of PB1-F2.

### PB1-F2 N66S decreases IFN activation in the context of viral infection

**Figure 2:**
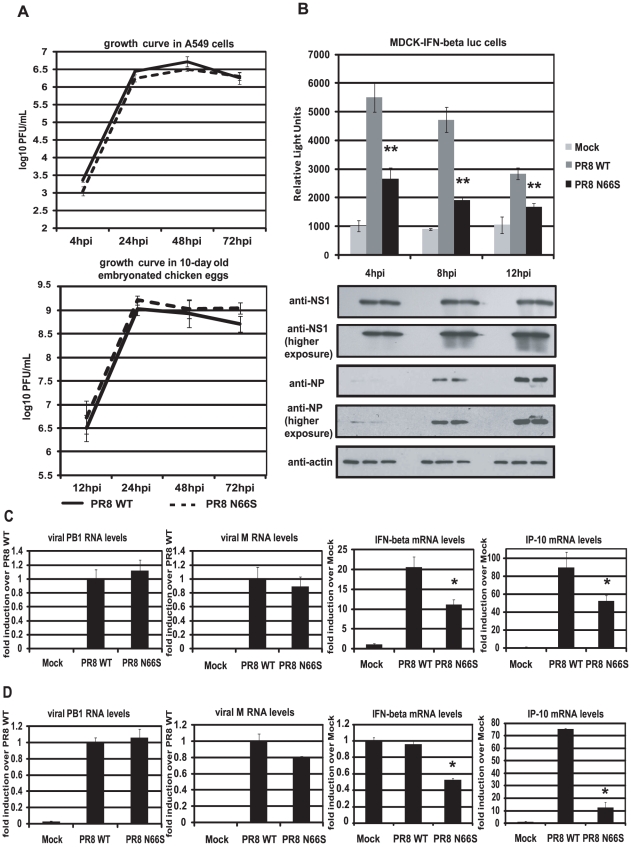
PB1-F2 N66S induces less IFN compared to PB1-F2 WT in the context of viral infection. (A) Growth curves of PR8 WT and PR8 N66S viruses in A549 cells and *in ovo*. A549 cells were infected with virus at an MOI of 0.01 and aliquots of the supernatants were taken at 4, 24, 48 and 72 hpi. To determine viral titers, supernatants were plaqued on MDCK cells. Ten-day old embryonated chicken eggs were inoculated with 100 PFU of virus and placed at 4°C at 12, 24, 48 and 72 hpi. The following days, allantoic fluids were harvested, spun down and titered via plaque assays on MDCK cells. (B) An MDCK cell line expressing an IFN-β promoter driven luciferase reporter (MDCK-IFN-beta luc) was infected with PR8 WT and PR8 N66S viruses at an MOI of 2. Cells were lysed at 4, 8 and 12 hpi and luciferase activity was measured. In addition, cell lysates were analyzed for protein expression via Western blots. (C and D) Primary murine dendritic cells (C) or murine lung epithelial cells (D) were infected with the indicated PR8 viruses at an MOI of 2. RNA was isolated 8 hpi and subjected to qRT-PCR. All data represent means ± standard deviations of one representative experiment (n = 3). Statistical significance was determined using Student's *t* test. *, p<0.05; **, p<0.01. Statistical significance is relative to PR8 WT. All data shown are representatives of at least two independent experiments.

To investigate whether PB1-F2 66S is a stronger IFN antagonist than PB1-F2 66N in the context of influenza virus infection, we employed reverse genetics to generate a PR8 influenza virus expressing PB1-F2 66S or 66N (WT). We then examined the growth kinetics of these two viruses in A549 cells, a human lung epithelial cell line which supports efficient viral replication, and in 10-day old embryonated chicken eggs. Both viruses displayed similar replication kinetics in both A549 cells and *in ovo* (Figure 2A). We next examined IFN induction by these two viruses at early timepoints of infection. For this purpose we infected an MDCK IFN-β reporter cell line with the PR8 viruses either expressing PB1-F2 66N or 66S. At 4 hours post infection (hpi), PR8 WT virus induced the IFN-β reporter approximately 5-fold over mock infected cells. This weak induction is due to the strong IFN antagonist activity of the NS1 protein. Yet, PR8 N66S suppressed the IFN-β reporter by 2-fold compared to the PR8 WT virus (Figure 2B). At 8 and 12 hpi similar results were observed. Of note, this phenomenon was not due to increased PB1-F2 protein expression by the PR8 N66S virus (data not shown). NS1 and NP proteins from both viruses were expressed at equal levels with increasing protein expression over time, as determined by Western blot analysis, indicating that the enhanced IFN suppression activity by the PR8 N66S virus was not due to increased replication in these cells or higher NS1 protein expression (Figure 2B). In Western blots with higher exposure, NP protein levels are visible at 4 hpi showing similar levels in PR8 WT and PR8 N66S virus infected cells (Figure 2B).

### The IFN antagonism function of PB1-F2 N66S is not cell-type specific

It has been suggested that PB1-F2 exerts its pro-apoptotic function specifically in immune cells [2]. We thus examined the IFN antagonism function of PB1-F2 N66S in both epithelial as well as immune cells. Murine dendritic cells were infected with PR8 viruses expressing either 66N or 66S PB1-F2 at an MOI of 2 and RNA was harvested 8 hpi to analyze gene expression via qRT-PCR. Infection with PR8 WT virus induced IFN-β mRNA levels approximately 20-fold over mock (Figure 2C). Infection with PR8 N66S, in contrast, induced two-fold less IFN-β (Figure 2C). We also examined mRNA levels of IP-10, an ISG, which showed a similar trend to the IFN-β induction by the two PR8 viruses (Figure 2C).

We also infected murine lung epithelial (LA-4) cells with the PR8 viruses and quantified IFN-β as well as IP-10 mRNA levels. Infection with PR8 WT virus did not efficiently increase IFN-β in these cells over mock treated cells, but we observed a two-fold suppression of IFN-β induction by the PR8 N66S virus (Figure 2D). In LA-4 cells, there was a more pronounced suppression of IP-10 mRNA levels compared to dendritic cells (Figure 2C and 2D).

### PB1-F2 N66S expressed from an NDV vector suppresses the induction of IFN in human dendritic cells

**Figure 3:**
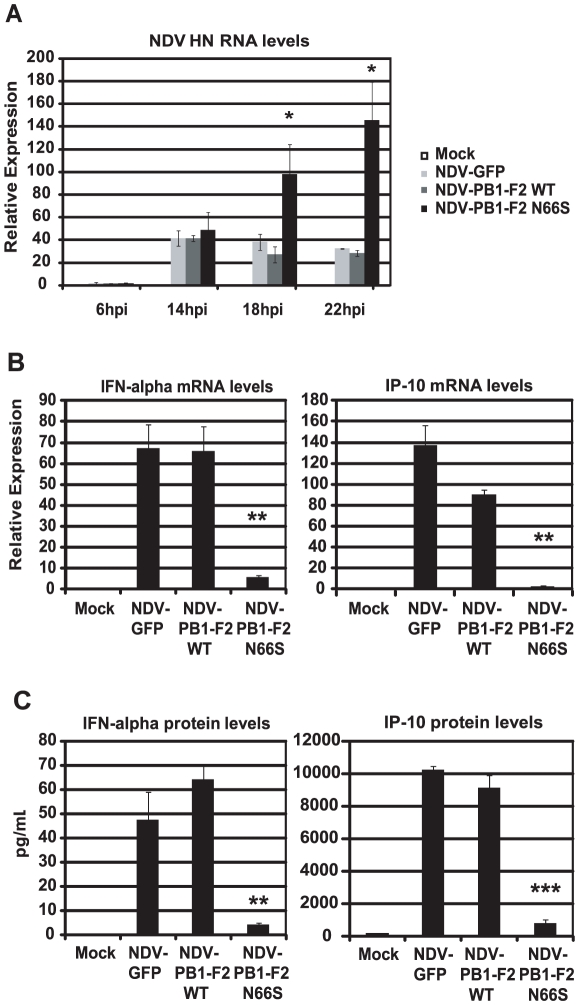
PB1-F2 N66S inhibits NDV induced IFN in primary human dendritic cells. (A) Primary human dendritic cells were infected with NDV-GFP, NDV-PB1-F2 WT or NDV-PB1-F2 N66S at an MOI of 2 and NDV HN was determined via qRT-PCR at 6,14,18 and 22 hpi. (B and C) At 14 hpi, qRT-PCR analyses were performed for IFN-alpha and IP-10 mRNA levels. In addition, supernatants were collected for ELISA analyses to quantify IFN-α and IP-10 protein secretion. All data represent means ± standard deviations of one representative experiment (n = 3). Statistical significance was determined using Student's *t* test. *, p<0.05; **, p<0.01; ***, p<0.001. Statistical significance is relative to NDV-GFP. The data presented in B and C are representatives of five independent experiments with different donors.

Newcastle disease virus (NDV) has been shown to be a potent IFN inducer by activating RIG-I [42]. Therefore, we asked the question whether PB1-F2 could block NDV induced IFN activation in primary human dendritic cells. Dendritic cells are important immune effector cells that are involved in clearing viral infections by secreting pro-inflammatory cytokines as well as type I IFN and bridging the innate and adaptive immune responses. It has been demonstrated that primary human dendritic cells are a suitable *ex vivo* model to study human influenza A virus host responses [43]. We generated recombinant NDV viruses that express GFP or PB1-F2 (WT and N66S) and infected primary human dendritic cells to investigate the induction of type I IFN in this model. As shown in Figure 3A, NDV-PB1-F2 N66S replicated more efficiently than the NDV-PB1-F2 WT and NDV-GFP viruses at 18 hpi and 22 hpi. To test whether this growth advantage may be due to suppressed IFN induction by PB1-F2 N66S expressed from the NDV vector, we analyzed IFN induction at 14 hpi by the three recombinant NDV viruses when replication was still found to be similar. NDV-GFP induced high levels of IFN as well as IP-10 as measured by qRT-PCR and ELISA (Figure 3B and 3C). Expression of PB1-F2 WT did not suppress IFN and IP-10 levels compared to GFP, whereas PB1-F2 N66S efficiently inhibited IFN induction in NDV infected dendritic cells (Figure 3B and 3C). These results indicate that PB1-F2 N66S is also able to suppress IFN production in primary human dendritic cells confirming that this inhibitory effect is not strictly cell type-specific.

### PB1-F2 N66S decreases IFN induction in the presence of an NS1 mutant deficient in dsRNA/TRIM25 binding

**Figure 4:**
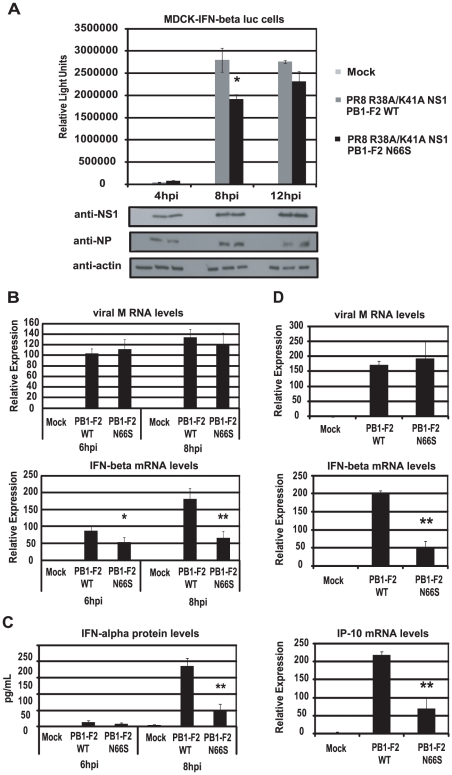
PB1-F2 N66S reduces IFN induction by a dsRNA and TRIM25 binding mutant NS1 influenza virus. (A) An MDCK cell line expressing an IFN-β promoter driven luciferase reporter (MDCK-IFN-beta luc) was infected with PR8 viruses expressing the dsRNA/TRIM25 binding mutant form of NS1 (R38A/K41A) as well as PB1-F2 WT or PB1-F2 N66S at an MOI of 0.5. Cells were lysed at 4, 8 and 12 hpi and luciferase activity was measured. In addition, cell lysates were analyzed for protein expression via Western blot analyses. (B and C) Primary human dendritic cells were infected with the viruses described in (A) at an MOI of 0.5 and RNA was harvested for qRT-PCR analyses at 6 and 8 hpi (B). In addition, supernatants were analyzed for IFN-alpha secretion via ELISA (C). (D) A549 cells were infected with the viruses described in (A) at an MOI of 0.5 and RNA was analyzed via qRT-PCR at 6 hpi. All data represent means ± standard deviations of one representative experiment (n = 3). Statistical significance was determined using Student's *t* test. *, p<0.05; **, p<0.01. Statistical significance is relative to PB1-F2 WT. Data shown are representatives of two independent experiments.

The NS1 protein is the major IFN antagonist of influenza viruses as the knockout of NS1 strongly attenuates the virus in IFN competent cells [44]. NS1 employs multiple strategies to counteract the initiation and establishment of an antiviral state in virus infected cells [45]. One of these strategies is the masking of viral RNA species from recognition by RIG-I [16], [17]. Within the N-terminal domain of NS1, the basic residues R38 and K41 were found to mediate binding to dsRNA species [16] and mutating these residues to alanine was shown to induce a robust IFN response in influenza virus infected cells [15]. In addition, these mutations also result in loss of NS1 interaction with TRIM25, an interaction that is required for inhibition of RIG-I activation mediated by this E3 ligase [20]. We thus aimed to examine the IFN induction by viruses that express a dsRNA/TRIM25 binding mutant form of NS1 and PB1-F2 66N or 66S. For this purpose we rescued PR8 viruses with the described point mutations in NS1 (R38A/K41A) and those that express the WT or N66S form of PB1-F2. Infection of the MDCK-IFN-β reporter cell line with these two mutant viruses resulted in approximately 3 log higher induction of the IFN-β reporter compared to the PR8 viruses with WT NS1 at 8 hpi (Figure 2B and 4A). At 4 hpi, no remarkable difference in IFN induction was seen between the PB1-F2 66N or 66S expressing viruses. At 8 hpi, however, the NS1 dsRNA/TRIM25 binding mutant virus expressing PB1-F2 66S induced significantly less IFN than the isogenic virus expressing PB1-F2 66N (Figure 4A). At 12 hpi, the difference in IFN induction became minimal (Figure 4A) and a substantial detachment of cells was observed at this time point (data not shown). At 4 hpi and 8 hpi, the NP and NS1 protein levels were comparable in cells infected with both viruses. Of note, the NP protein levels in cells infected with the NS1 mutant viruses do not increase as dramatically between 4 and 8 hpi compared to those in cells infected with PR8 viruses expressing wildtype NS1 (Figure 2B). This finding indicates that the NS1 dsRNA binding mutant viruses are attenuated compared to the wildtype NS1 expressing viruses. Next, we infected primary human dendritic cells with the NS1 dsRNA/TRIM25 binding mutant viruses and analyzed the induction of type I IFN via qRT-PCR and ELISA. At 6 hpi and 8 hpi, IFN-β mRNA as well as IFN-α protein levels were decreased by the PB1-F2 N66S expressing virus compared to the isogenic virus (Figure 4B and 4C) which confirms the data obtained in the MDCK IFN-β reporter cell line (Figure 4A). We also performed qRT-PCR analyses to quantify IFN-α mRNA levels and found a similar trend to IFN-β mRNA levels (data not shown). To test this phenomenon in epithelial cells, we also infected A549 cells with the dsRNA/TRIM25 binding mutant NS1 viruses. As shown in Figure 4D, the PB1-F2 N66S expressing virus induced less IFN and IP-10 compared to the PB1-F2 WT virus at 6 hpi and 8 hpi (data not shown). In summary, these data demonstrate that even in the presence of an NS1 protein that lacks the dsRNA and TRIM25 binding function, PB1-F2 66S is able to reduce the induction of IFN compared to PB1-F2 66N.

### PB1-F2 N66S enhances NS1 mediated IFN antagonism

**Figure 5:**
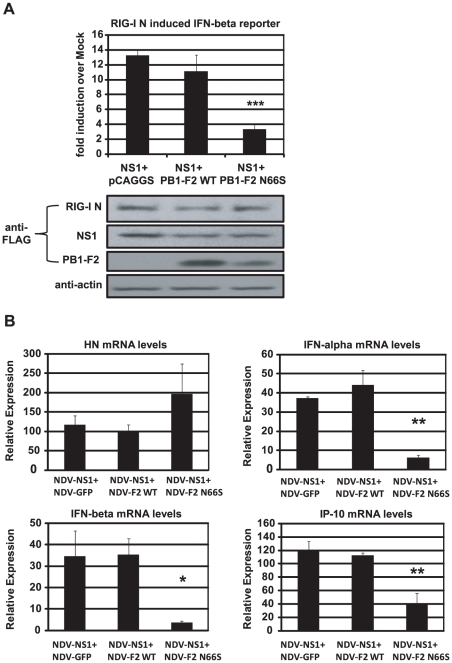
PB1-F2 N66S further enhances NS1 mediated IFN antagonism in an overexpression system. (A) 293T cells were transfected with IFN-β reporter, renilla reporter, RIG-I N expression plasmid and the indicated combination of expression plasmids. Twenty-four hours post transfection, luciferase and renilla signals were determined by luminometry. In addition, Western blot analyses were applied to determine protein expression levels from cell extracts. (B) Primary human dendritic cells were co-infected with the indicated combination of recombinant NDV viruses at an MOI of 1 per virus. RNA was harvested and subjected to qRT-PCR 14 hpi. All data represent means ± standard deviations of one representative experiment (n = 3). Statistical significance was determined using Student's *t* test. *, p<0.05; **, p<0.01; ***, p<0.001. Statistical significance is relative to NS1+pCAGGS and NDV-NS1+NDV-GFP. Data presented in A are representatives of two independent experiments and data in B are representatives of three independent experiments performed in different donors.

To characterize the relationship between NS1 and PB1-F2 regarding IFN antagonism, we overexpressed NS1 with either PB1-F2 66N or 66S in 293T cells and analyzed the induction of the IFN-β reporter activated by RIG-I N. As shown in Figure 5A, PB1-F2 66N did not significantly reduce IFN promoter activation compared to the empty vector control when co-expressed with NS1. In contrast, PB1-F2 66S further decreased IFN induction when co-expressed with NS1 even though PB1-F2 66N is expressed more efficiently as shown in Western blot analyses (Figure 5A). To confirm these findings in the human DC model, we performed a co-infection experiment with the recombinant NDV viruses. Co-infection of human DCs with NDV-NS1 and NDV-PB1-F2 66N induced similar levels of IFN compared to co-infection with NDV-NS1 and NDV-GFP (Figure 5B). In contrast, co-infection of primary human DCs with NDV-NS1 and NDV-PB1-F2 N66S strongly reduced type I IFN and IP-10 mRNA levels (Figure 5B). Collectively, these data indicate that PB1-F2 N66S, but not PB1-F2 WT, enhances the IFN antagonism activity of NS1.

### PB1-F2 inhibits the induction of IFN at the level of MAVS

**Figure 6:**
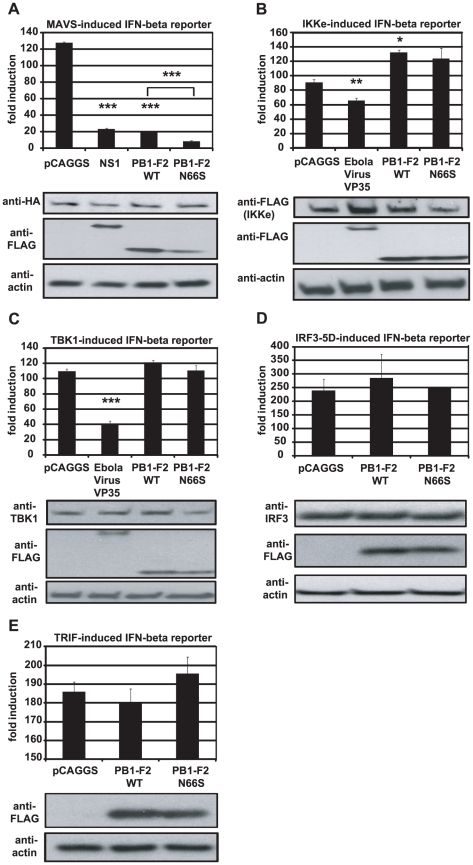
PB1-F2 inhibits IFN induction at the level of MAVS. (A–E). 293T cells were transfected with an IFN-β reporter, a renilla reporter and one of the following to induce the IFN-β reporter: MAVS-HA (A), IKKε-FLAG (B), TBK1-FLAG (C), IRF3-5D (D) or TRIF-FLAG (E) expression plasmids. Also, pCAGGS, expression plasmids for PB1-F2 WT or N66S or a positive control (NS1 or VP35) were transfected. Twenty-four hours post transfection, lysates were harvested for luminometry and Western blot analyses. The unit on the y-axis indicates fold induction over Mock. All data represent means ± standard deviations of one representative experiment (n = 3). Statistical significance was determined using Student's *t* test. *, p<0.05; **, p<0.01; ***, p<0.001. All data shown are representatives of at least two independent experiments.

In order to examine at which stage the PB1-F2 protein inhibits the IFN production pathway, we used different stimuli downstream of RIG-I to induce the IFN-β reporter in 293T cells. We overexpressed MAVS, TBK1, IKKε and IRF3-5D, a phosphomimetic form of IRF3, and analyzed the activation of the IFN-β reporter in the presence of PB1-F2. We observed that PB1-F2 WT and N66S inhibited the MAVS-induced IFN-β reporter, but did not affect any factors downstream of MAVS such as TBK1 or IRF3-5D (Figure 6A, 6C, 6D). We performed densitometry analyses and found that the MAVS expression levels were not decreased in NS1, PB1-F2 WT or PB1-F2 N66S expressing cells compared to the empty vector control (data not shown). Upon stimulation with IKKε, we found a slight enhancement of the IFN-β reporter activity in cells expressing PB1-F2 WT (Figure 6B). For TBK1 and IKKε (Figure 6B and 6C), Ebola virus VP35 protein was used as a positive control since it has been previously described by Prins *et al.* to bind to both kinases and thus interfere with the phosphorylation of IRF3 [13]. NS1 has been reported to inhibit MAVS in an IFN-β reporter assay by a yet unclear mechanism [46]. To confirm that PB1-F2 inhibits IFN production at the level of MAVS, we tested whether PB1-F2 affects a TRIF-induced IFN-β reporter. TRIF is an adaptor protein that mediates IFN production upon activation of endosomal TLRs in a MAVS-independent manner. As shown in Figure 6E, the PB1-F2 proteins did not affect TRIF-induced IFN which indicates that PB1-F2 inhibits IFN in a MAVS-dependent fashion.

**Figure 7:**
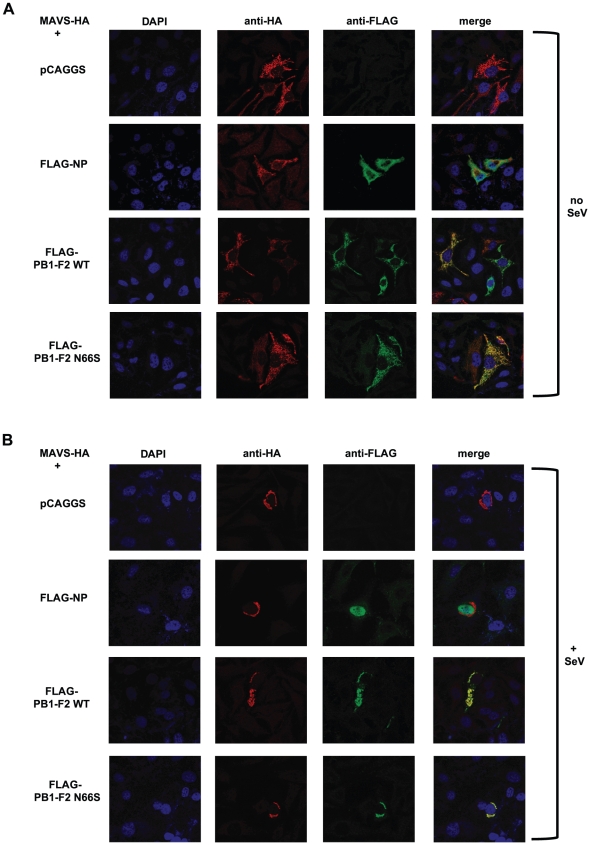
PB1-F2 co-localizes with MAVS. (A and B) Hela cells were transfected with MAVS-HA expression plasmid along with pCAGGS alone or pCAGGS expressing FLAG-NP, FLAG-PB1-F2 WT or N66S. Twenty-four hours post transfection, cells were fixed with 4% para-formaldehyde (A) or infected with Sendai virus for 18 hours and subsequently fixed (B). Fixed cells were stained with anti-FLAG and anti-HA antibodies as well as DAPI. Secondary anti-mouse antibodies conjugated to Alexa 555 and 488 were used to visualize stained proteins. Samples were inspected via confocal microscopy at a magnification of 100×. Images are representatives of three replicates. The images shown are representatives of at least two independent experiments.

Next, we investigated whether PB1-F2 co-localizes with MAVS at the mitochondria to confirm the reporter assay data shown in Figure 6A. For this purpose we overexpressed HA-tagged MAVS and FLAG-tagged PB1-F2 proteins in Hela cells and examined localization patterns via confocal microscopy. We chose Hela cells for our studies because they have been previously used to investigate MAVS localization upon influenza virus infection [47]. As shown in Figure 7A, both PB1-F2 66N and 66S co-localized with MAVS while the influenza virus nucleoprotein (NP), which does not affect IFN induction (data not shown), did not. It has been reported that MAVS undergoes a redistribution in the mitochondrial membrane upon activation of the IFN pathway [47]. We thus asked whether PB1-F2 could also associate with redistributed MAVS. Upon infection of transfected Hela cells with Sendai virus (SeV), we observed a relocalization of MAVS into distinct speckle-like structures (Figure 7B) as has been shown previously by Onoguchi *et al.* [47]. Interestingly, PB1-F2 also co-localized with these structures, while NP did not appear to do so (Figure 7B). In summary, these data indicate that PB1-F2 inhibits the induction of IFN at the level of MAVS, possibly by interacting with MAVS and/or affecting MAVS function.

## Discussion

In our previous work, we have shown that infection of mice with a recombinant influenza virus expressing PB1-F2 of the H5N1 Hong Kong 1997 strain with an N66S mutation results in decreased IFN induction in the lungs compared to infection with the isogenic wildtype virus without affecting the levels of the induction of apoptosis [5]. Here, we report on the molecular mechanism by which an N66S point mutation in the PR8 PB1-F2 protein leads to decreased IFN induction in both overexpression as well as viral infection models (Figure 1 and 2). We show that PB1-F2 wildtype (66N) has IFN antagonism activity in a RIG-I N induced IFN-β reporter assay and an NDV-GFP bioassay when overexpressed in 293T cells. However, the anti-interferon function of PB1-F2 66N seems to be weaker than that of NS1 or PB1-F2 66S in a 293T cell overexpression system (Figure 1A and 1B). In human dendritic cells, PB1-F2 66N shows no to minimal IFN antagonism activity compared to GFP when expressed from an NDV vector that induces high levels of IFN. This may be due to a diminished IFN antagonism activity of the PB1-F2 WT protein in immune cells (Figure 3). These results indicate that PB1-F2 66N is a weaker IFN antagonist than NS1 or PB1-F2 66S in all systems studied. Interestingly, PR8 N66S virus suppressed IP-10 mRNA levels to a greater extent in epithelial cells than in dendritic cells (Figure 2C and 2D) which may have to do with a differential regulation of IP-10 expression in these cell types.

We also tested the PB1-F2 proteins of the 1918 pandemic and the A/Viet Nam/1203/2004 (H5N1) viruses and found an inhibition of RIG-I N induced IFN-β reporter in 293T cells (data not shown). We thus believe that the IFN antagonism activity of PB1-F2 is a general function shared by various influenza A virus strains. The pro-apoptotic activity was found to be mediated by the C-terminal domain of PB1-F2 which interacts with VDAC1 and ANT3 [26]. Interestingly, we also found that this C-terminal region spanning amino acids 38 to 87 was sufficient to inhibit the induction of IFN (Figure 1D). Further work will focus on characterizing the minimal region needed to suppress IFN and identifying key residues mediating the IFN antagonism function of PB1-F2. Also, it will be important to determine the relationship between the pro-apoptotic and anti-interferon function of PB1-F2. A recent report describes a possible anti-apoptotic role of MAVS by interacting with and destabilizing VDAC1 [48]. PB1-F2 might interfere with the interaction between MAVS and VDAC1 and thus decrease IFN production while promoting VDAC1-mediated cell death. Further work will be necessary to test this hypothesis.

Of note, a recent report by Le Goffic *et al.* indicates that PB1-F2 enhances the induction of IFN in epithelial cells [49]. In agreement with the findings of Le Goffic *et al.*, we observed decreased IFN levels of a PR8 virus lacking PB1-F2 compared to wildtype PR8 virus. Further characterization of the PB1-F2 knockout virus revealed increased levels of the N40 protein in infected cells, while N40 protein levels expressed by the PB1-F2 N66S virus were similar to that found in wildtype virus infected cells (data not shown). The increased N40 expression levels in PB1-F2 (PR8) knockout virus infected cells is in accordance with a report by Wise *et al.* [50]. N40 is a third protein expressed from the PB1 gene and has been identified as a truncated form of PB1 [50]. It is possible that the reduced IFN levels caused by PB1-F2 knockout viruses are due to altered N40 expression levels and we thus feel that the use of a PB1-F2 knockout virus is not suitable to assess the induction of IFN.

Despite the reduced induction of IFN by PB1-F2 N66S, we do not observe a growth advantage of the PR8 PB1-F2 N66S virus in A549 cells or embryonated chicken eggs (Figure 2A). It is possible that moderate differences in IFN levels induced by the virus do not affect viral replication in a cell line that is highly adapted to support influenza virus growth. PB1-F2 N66S may confer a growth advantage in cells where NS1 may be non-functional or restricted in its IFN antagonism capability. In fact, we have shown that a virus containing mutations that render NS1 deficient in dsRNA and TRIM25 binding induces high levels of IFN and PB1-F2 66S is able to reduce the induction of IFN by this virus compared to PB1-F2 66N (Figure 4). Furthermore, overexpression of PB1-F2 N66S in combination with NS1 further enhances the anti-IFN activity of NS1 (Figure 5). PB1-F2 N66S may target a protein that is not affected by NS1 or support NS1 in inhibiting a particular factor important for IFN production.

We found that PB1-F2 inhibits a MAVS-induced IFN-β reporter, but doesn't affect the reporter when induced by factors that are downstream in the IFN production pathway, namely the IRF3 kinases or IRF3 (Figure 6A–6D). Further work will be necessary to identify the domain of MAVS and/or adaptor proteins targeted by PB1-F2.

The question arises how a mutation from an asparagine to serine at position 66 could increase the IFN antagonism function of PB1-F2. This residue lies within the minimal mitochondrial targeting sequence of PB1-F2 [51] and it is possible that an N66S mutation alters the efficiency of mitochondrial targeting, potentially by altering the secondary structure. This may allow for more efficient interactions with MAVS and/or insertion into the mitochondrial membrane to disrupt MAVS function, for example by interfering with the interactions of MAVS with adaptor proteins. Alternatively, an N66S mutation could create a phosphorylation site that is now recognized by cellular kinases.

Multiple IFN antagonists of influenza virus have been described (NS1, PB1, PB2, PA, PB1-F2) [5], [23], [24], [44] which demonstrates that the IFN antagonism strategies used by influenza virus are more complex than previously thought. It will be important to study and understand the biological significance for this functional redundancy. In this report, we have characterized the IFN antagonism function of PB1-F2 and provide evidence that PB1-F2 N66S works in conjunction with NS1. Further work will be needed to understand the interplay between PB1-F2 and the polymerase proteins regarding IFN antagonism. Overall, our findings contribute to understanding the molecular mechanism for PB1-F2 mediated pathogenicity and highlight the importance of position 66 in the PB1-F2 protein for virulence.
